# LinkHub: a Semantic Web system that facilitates cross-database queries and information retrieval in proteomics

**DOI:** 10.1186/1471-2105-8-S3-S5

**Published:** 2007-05-09

**Authors:** Andrew K Smith, Kei-Hoi Cheung, Kevin Y Yip, Martin Schultz, Mark B Gerstein

**Affiliations:** 1 Department of Molecular Biophysics and Biochemistry, Yale University, New Haven, Connecticut, 06520, USA; 2 Department of Computer Science, Yale University, New Haven, Connecticut, 06520, USA; 3 Center for Medical Informatics, Yale University, New Haven, Connecticut, 06520, USA; 4 Department of Genetics, Yale University, New Haven, Connecticut, 06520, USA; 5 Program in Computational Biology and Bioinformatics, Yale University, New Haven, Connecticut, 06511, USA; 6 Department of Anesthesiology, Yale University, New Haven, Connecticut, 06520, USA

## Abstract

**Background:**

A key abstraction in representing proteomics knowledge is the notion of unique identifiers for individual entities (e.g. proteins) and the massive graph of relationships among them. These relationships are sometimes simple (e.g. synonyms) but are often more complex (e.g. one-to-many relationships in protein family membership).

**Results:**

We have built a software system called LinkHub using Semantic Web RDF that manages the graph of identifier relationships and allows exploration with a variety of interfaces. For efficiency, we also provide relational-database access and translation between the relational and RDF versions. LinkHub is practically useful in creating small, local hubs on common topics and then connecting these to major portals in a federated architecture; we have used LinkHub to establish such a relationship between UniProt and the North East Structural Genomics Consortium. LinkHub also facilitates queries and access to information and documents related to identifiers spread across multiple databases, acting as "connecting glue" between different identifier spaces. We demonstrate this with example queries discovering "interologs" of yeast protein interactions in the worm and exploring the relationship between gene essentiality and pseudogene content. We also show how "protein family based" retrieval of documents can be achieved. LinkHub is available at hub.gersteinlab.org and hub.nesg.org with supplement, database models and full-source code.

**Conclusion:**

LinkHub leverages Semantic Web standards-based integrated data to provide novel information retrieval to identifier-related documents through relational graph queries, simplifies and manages connections to major hubs such as UniProt, and provides useful interactive and query interfaces for exploring the integrated data.

## Background

Biological research is producing vast amounts of data (e.g. from high throughput experiments such as sequencing projects, and microarray experiments) at a prodigious rate. Most of this data is made freely available to the public, and this has created a large and growing number of internet and world wide web-accessible biological data resources which are characterized by being distributed, heterogeneous, and having a large size variance, i.e. huge, mega-databases such as UniProt [1] down to medium, small or "boutique" databases (e.g., TRIPLES [2]) generated for medium or small scale experiments or particular purposes. Most computational analyses of biological data will require using multiple integrated datasets, and integrated data along with rich, flexible and efficient interfaces to it encourages exploratory data analysis which can lead to serendipitous new discoveries: the sum is greater than its parts. Currently, integration often must be done manually in a labor and time intensive way by finding relevant datasets, understanding them, writing code to combine them, and finally doing the desired analysis. The basic requirements for better, more seamless integrated analysis are uniformity and accessibility; data are ineffectual if scattered among incompatible resources.

Web search engines and hyperlinks are the basic and commonly used ways to find things on the web and navigate web content but they do not enable fine-grained cross-site analysis of data. To improve upon this, one key issue is the need for standardization and its widespread use, and tools supporting and enabling it. Biological data is too vast for brute-force centralization to be the complete solution to data interoperability. We must have standards and systems for people and groups to work independently creating and making data available (although ultimately cooperatively and collaboratively) but still in the end all or most of the pieces of biological knowledge and data are connected together in semantically rich ways. The W3C's [3]*Semantic Web *[4-6] is a promising candidate: it allows web information to be expressed in fine-grained structured ways so applications can more readily and precisely extract and cross-reference key facts and information from it without having to worry about disambiguating meaning from natural language texts. Standard and machine-readable ontologies such as the Gene Ontology [7] are also created and their common use encouraged to further reduce semantic ambiguity, although there is a need to make these ontologies more machine-friendly [8].

**Figure 1:**
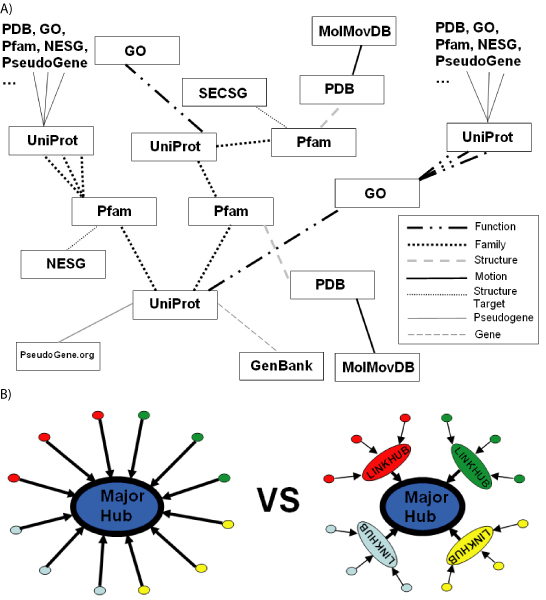
**Graph of relationships and hub of hubs organization**. (a) A conceptualization of the semantic graph of interrelationships among biological identifiers, with boxes representing instances of biological identifiers (originating database names given inside) and different edge types representing different kinds of relationships (b) LinkHub as an enabler of an efficient "hub of hubs" organization of biological data. The different colors represent different labs, organizations, or logical groupings of data resources.

A key abstraction or "scaffold" for representing biological data is the notion of unique identifiers for biological entities and relationships among them. For example, each protein sequence in the UniProt database is given a unique accession, e.g. Q60996, which can be used as a key to access its UniProt sequence record. UniProt sequence records also contain cross-references to related information in other databases, e.g. related Gene Ontology and PFAM identifiers (although the relationship types, e.g. "functional annotation" and "family membership" respectively, are not specified). Two identifiers such as Q60996 and GO:0005634 and the cross-reference between them can be viewed as a single edge between two nodes in a graph, and conceptually then a large, important part of biological knowledge can be viewed as a massive graph whose nodes are biological entities such as proteins, genes, etc. represented by identifiers and the links in the graph are typed and are the specific relationships among the biological entities. Figure 1a is a conceptual illustration of the graph of biological identifier relationships; the problem is that this graph only concretely exists piecemeal or not at all.

A basic problem preventing this graph of relationships from being more fully realized is the problem of nomenclature. Often, there are many synonyms for the same underlying entity caused by independent naming, e.g. structural genomics centers assigning their own protein identifiers in addition to UniProt's. There can also be lexical variants of the same underlying identifier (e.g. GO:0008150 vs. GO0008150 vs. GO-8150). Synonyms are a small part of the overall problem, however, and more generally there are many kinds of relationships including one-to-one and one-to-many relationships. For example, a single Gene Ontology or PFAM identifier can be related with many UniProt identifiers (i.e. they all share the same functional annotation or family membership). PFAM represents an important structuring principle for proteins and the genes they come from, the notion of families (or domains) based on evolution; proteins sharing common PFAM domains are evolutionarily related (called *homologs*) and likely have the same or similar functions. Gene Ontology consists of three widely used structured, controlled vocabularies (ontologies) that describe gene products such as proteins in terms of their associated biological processes, cellular components and molecular functions in a species-independent manner. The conceptual graph of identifier relationships is richly connected, and a transitive closure even a few levels deep can lead to indirect relationships with a great number of other entities. Being able to store, manage, and work with this graph of entities and relationships can lead to many opportunities for interesting exploratory analysis and LinkHub is such a system for doing this.

### LinkHub: a system for loosely coupled, collaborative integration of biological identifier relationships

**Figure 2:**
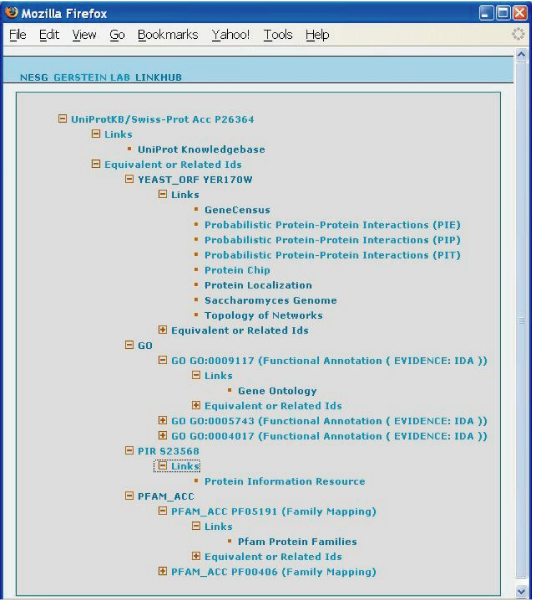
**The basic DHTML list interface to LinkHub**. Here, the data and relationships for UniProt identifier P26364 are presented. P26364 is presented at the root of the list, and lower levels contain information on additional related identifiers. Each identifier has two subsections: Links which gives a list of hyperlinks to web documents directly relevant to the identifier; and Equivalent or Related Ids which contains a list of additional identifiers related to the first identifier (the relationship type if it exists is given in parentheses; a synonym relationship is assumed if no relationship is given). The identifiers in the Equivalent and Related Ids section may themselves be further related to other identifiers which will have their own Links and Equivalent or Related Ids sections, ad nauseum. The initial display shows the transitive closure of the root identifier one level deep, and dynamic callbacks to the server retrieve additional data when the user clicks on identifiers whose subsections have not yet been loaded; in this way, the user can explore the relationship paths he desires without performance penalties (of loading the whole graph) or 'information overload'. The interface is dynamic, and a '+' list icon can be expanded to view the hidden underlying content, and a '-' list icon can be clicked to hide the content.

The Semantic Web is increasingly gaining traction as the key standards-based platform for biological data integration [9,10], and since LinkHub is a natural fit to Semantic Web technologies we use them as the basis of LinkHub. LinkHub is designed based on a semantic graph model, which captures the graph of relationships among biological entities discussed above. To provide a scalable implementation while also exploring Semantic Web database technologies, we implemented LinkHub in both MySQL [11] and Resource Description Framework or RDF [12] databases. LinkHub provides various interfaces to interact with this graph, such as a web frontend for viewing and traversing the graph as a dynamic expandable/collapsible HTML list (see figure 2) and a mechanism for viewing particular path types in the graph, as well as with RDF query languages.

Centralized data integration to an extent does make sense, e.g. a lab or organization might want to create a local data warehouse of interconnections among its individual data resources; but it does not want to have to explicitly connect its data resources up to everything in existence, which is impossible. The key idea is that if groups independently maintaining data resources each connect their resources up to some other resource X, then any of them can reach any other through these connections to X, and we can collectively achieve incremental global data integration in this way. LinkHub is a software architecture and system which aims to help realize this goal by enabling one to create such local minor hubs of data interconnections and connect them to major hubs such as UniProt in a federated "hub of hubs" framework and this is illustrated in figure 1b.

### Paper organization

In the results section next, we will demonstrate how LinkHub enables novel information retrieval to documents attached to LinkHub graph nodes based on the relational structure of the LinkHub graph; a particular practical use case of this, providing "family views" to data, will be given. We will then give concrete examples of the kinds of integrated, cross-database queries that can be done with LinkHub, in combination with a previous system of ours called YeastHub, in support of scientific exploratory analysis; example queries discovering "interologs" of yeast protein interactions in the worm and exploring the relationship between gene essentiality and pseudogene content will be given. We will then discuss related work to LinkHub and future directions before concluding. In the methods section we describe implementation details of LinkHub, including its data models and how they are populated with data and LinkHub's web interactive and query interfaces.

## Results

### Novel information retrieval based on LinkHub relational graph structure

The "path type" interface to LinkHub allows one to flexibly retrieve useful subsets of the web documents attached to identifier nodes in the graph based on the graph's relational structure. Normal search engines relying on keyword searches could not provide such access, and LinkHub thus enables novel information retrieval to its known web documents. An important practical use of this "path type" interface is as a secondary, orthogonal interface to other biological databases in order to provide different views of their underlying data. For example, MolMovDB [13] provides movie clips of likely 3D motions of proteins, and one can access it by PDB [14] identifiers. However, an alternative useful interface (actually provided by LinkHub) is a "family view" where one queries with a PDB identifier and can view all available motion pages for proteins in the same family as the query PDB identifier. LinkHub also provides a similar "family view" interface to structural genomics data in the SPINE system [15]. The system is flexible and one can easily imagine other similar applications, e.g. a "functional view" where all pages for proteins that have the same Gene Ontology function as a given protein are shown or a "pseudogene family view" where all pages for pseudogenes of proteins in the same family are shown. While the "path type" interface is a simple way of providing novel, relational access to LinkHub identifier node-linked documents, RDF query language access to the LinkHub relational graph would allow the most flexible novel information retrieval.

### Cross-database RDF queries

To demonstrate the data interaction and exploration capabilities engendered by the RDF version of LinkHub, the RDF-formatted LinkHub dataset is loaded into our YeastHub [16] system which uses Sesame [17] as its native RDF repository. Two demonstration queries below written in SeRQL (Sesame implementation of RQL) [18] demonstrate one can efficiently do the kinds of interesting preliminary scientific investigation and exploratory analysis commonly done at the beginning of research initiatives (e.g. to see whether they are worth pursuing further). These queries make use of information present in both YeastHub and LinkHub (and thus could not be done without joining the two systems), and LinkHub is used as 'glue' to provide connections (both direct and indirect) between different identifiers. It is noteworthy that these queries can be formulated and run in relatively little time (a few hours at most) and they roughly duplicate some results from published papers. In effect, LinkHub does the up-front time-consuming manual work of integrating multiple datasets, and this integrated data is generally useful for efficient formulation and execution of queries, which is in contrast to the papers which likely required extensive "one-off" effort to combine the necessary data to achieve their results.

### Query 1: finding worm 'interologs' of yeast protein interactions

**Figure 3:**
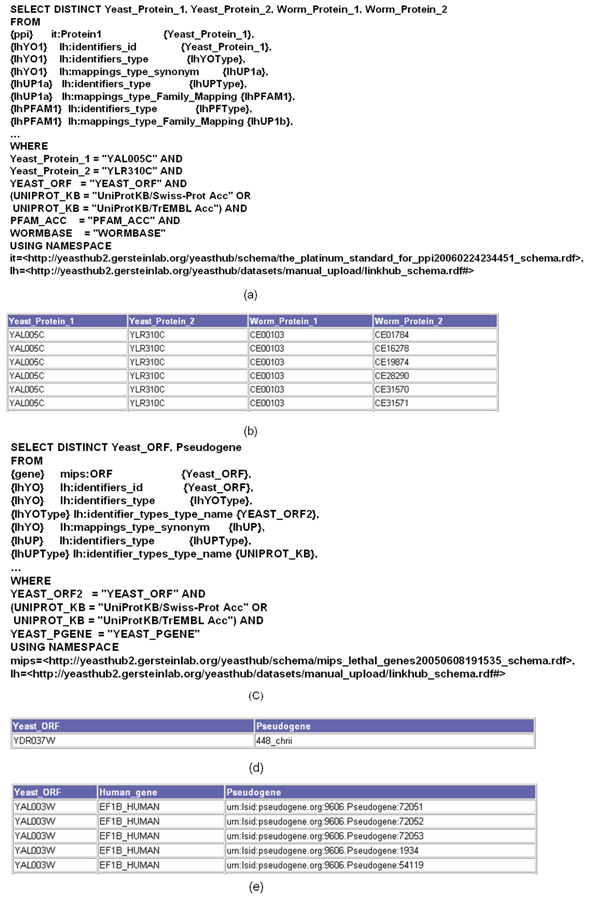
**Example RDF queries**. (a) shows a part of the SeRQL query that finds pairs of worm (C. elegans) proteins homologous to pairs of interacting proteins in yeast (C. cervisiae), i.e. "interologs". b) shows part of the corresponding query results. (c) shows the SeRQL query that explores the relationship between gene essentiality and the level of pseudogene content in yeast, which is one feature that might be hypothesized to be associated with essentiality, with queries of the joined YeastHub and LinkHub data. (d) shows the yeast pseudogenes found, interestingly only one. (e) shows part of the list of pseudogenes found in human homologs for a similar query; the full list is long, around 20000, consistent with there being many known pseudogenes in humans.

Proteins rarely act in isolation and often interact with one another and other molecules to perform necessary cellular actions. Experimental determinations of protein interactions are expensive and computational methods can leverage them for further interaction predictions. With this query we want to consider all the protein interactions in yeast (*S. cervisiae*) and see how many and which of them are present as evolutionarily related homologs in worm (*C. elegans*), also known as interologs [19], i.e. protein pairs in worm corresponding to evolutionarily related known interacting pairs in yeast. We thus start with a dataset containing known and predicted yeast protein interactions which is already loaded into YeastHub; here the interactions are expressed between yeast gene names. Part of the SeRQL statement for this query together with a portion of its corresponding output can be seen in figure 3. However, abstractly, the query is doing the following. For each yeast gene name in the interaction set we can use LinkHub's data as 'glue' to determine its homologs (via Pfam) in worm by traversing identifier type paths in the LinkHub relationship graph like the following:

yeast gene name → UniProt Accession → Pfam accession → UniProt Accession → WormBase ID.

Then, for each pair in the yeast protein interaction dataset, we determine if both of its yeast gene names lead to WormBase IDs [20] in this way and print those WormBase IDs as possible protein interactions if so.

### Query 2: exploring pseudogene content versus gene essentiality in yeast and humans

Pseudogenes are genomic DNA sequences similar to normal genes (and usually derived from them) but are not expressed into functional proteins; they are regarded as defunct relatives of functional genes [21,22]. In the queries here we explore the relationship between gene essentiality (a measure of how important a gene is to survival of an organism) and the number of pseudogenes in an organism. We might hypothesize that more essential genes might have larger numbers of pseudogenes, and we explore this idea with queries of the joined YeastHub and LinkHub data. First, YeastHub has the MIPS [23] Essential Genes dataset, and we use this as our data on gene essentiality; LinkHub contains a small dataset of yeast pseudogenes [24].

Abstractly, for each yeast gene name in the list of essential genes, we determine its pseudogenes by traversing identifier type paths in the LinkHub graph like the following:

yeast gene name → UniProt Accession → yeast pseudogene

For each essential yeast gene we then determine how many pseudogenes it has. We can then inspect the list of essential genes to see if there is a relationship between essentiality and number of pseudogenes. Humans have a large number of known pseudogenes [25] but gene essentiality is difficult to characterize in humans (with many tissue types and developmental states complicating the issue). Since essentiality is well studied in yeast, one thing we can do is determine the human homologs of yeast essential genes, which would perhaps likely be "more important" in a survival sense, and examine them for patterns associated with essentiality. For each yeast gene name in the list of essential genes, we can find the homologous pseudogenes in human by traversing identifier type paths in the LinkHub graph like the following:

yeast gene name → UniProt Accession → Pfam accession → human UniProt Id → UniProt Accession → Pseudogene LSID

Part of the SeRQL for the first query (for yeast pseudogenes) and results from both can be seen in figure 3, and they show that few yeast essential genes are associated with pseudogenes whereas this is not the case with human. This may reflect the difference in processes of creation of the predominate numbers of yeast and human pseudogenes (duplication vs retrotransposition, see [21,22]).

## Discussion

### Related work

The basic conceptual underpinnings of LinkHub, i.e., the importance of biological identifiers and linking them, was given by Karp [26]. LinkHub uses a Semantic Web approach to build a practical system based on and extending Karp's ideas on database links. The Semantic Web approach can also be used to implement database integration solutions based on the general approaches of *data warehousing *[27,28] and *federation *[29-31]. Essentially, data warehousing focuses on data translation, i.e. translating and combining multiple datasets into a single database, whereas federation focuses on query translation, i.e. translating and distributing the parts of a query across multiple distinct databases and collating their results into one. A methodological overview and comparison of these database integration approaches was discussed in the biomedical context [32]. LinkHub's architecture is a hybrid of these two approaches: individual LinkHub instantiations are a kind of mini, local data warehouse of commonly grouped data and these are connected to large major hubs such as UniProt in a federated fashion; efficiency is gained by obviating the need for all source datasets to be individually connected to the major hubs.

LinkHub differentiates itself by not integrating all aspects of biological data but rather focusing on an important and more manageable high-level structuring principal, namely biological identifiers and the relationships (and relationship types) among them; hyperlinks to identifier-specific pages present in the "Links" section of the LinkHub web interface give access to additional attributes and data. In fact, our YeastHub system addressed integration more generally by transforming many datasets to common RDF format and storing and giving RDF query access to them in an RDF database. The problem with YeastHub was that the integration was thin, with rich connections among the integrated datasets being limited. LinkHub is thus useful and complementary to YeastHub in this respect as a "connecting glue" among the datasets in that it makes and stores these cross-references and enables better integrated access to the YeastHub data; the example queries above demonstrated this.

LinkHub's primary web interface can be viewed as a kind of "Semantic Web browser". Other work has also attempted to build browsers for Semantic Web data, including HayStack [33], Sealife [34], and BioGuide [35]. LinkHub is a more lightweight browser than HayStack (with a focus on biological relationship browsing) and differs from Sealife by being data-centric (establishing semantic links between data identifiers while treating web documents as metadata associated with the identifiers) as opposed to document-centric (establishing semantic links between terms/phrases appeared in different web documents). BioGuide uses RDF similar to LinkHub, but it is limited in that it focuses on abstract conceptual modelling of resources and their interconnections rather than on instance data as LinkHub; also its interface presents the data using graph drawing software with Java, whereas LinkHub is more lightweight and relies only on the web browser with JavaScript. Finally, there have been a number of graph database systems and query languages developed through the years but they suffer from being proprietary; none have developed into widely used standard systems. However, it should be pointed out that some of these systems support advanced graph data mining and analysis operations not supported by RDF query languages and these features might be necessary for effective analysis of biological data represented in RDF [36].

### Future directions

Currently, LinkHub has limited web document hyperlinks attached to its nodes, and if this could be increased the utility of the novel information retrieval based on querying the LinkHub relational graph, e.g. "path type" interface, would be enhanced. We are working to leverage the rich information in the LinkHub relational graph for enhanced automated information retrieval to web or scientific literature (MedLine) documents relevant to identifier nodes, e.g. proteomics identifiers, in the graph. A simple search for the identifier itself would likely not give optimal results due to conflated senses of the identifier text and identifier synonyms. In general, we need to consider and query for the key related concepts of an identifier, and these are present in the LinkHub subgraph surrounding the identifier. We consider the web pages attached to the identifiers in the subgraph as a "gold standard" for what additional relevant documents should be like, and we plan to use them as training sets to construct classifiers used to score and rank additional documents for relevance. We feel that this idea could be generalized and that the Semantic Web, which provides detailed information about terms and their relationships, could be leveraged to provide enhanced automated information retrieval or web search for Semantic Web terms.

We also hope to explore how other relevant Semantic Web-related technologies could be effectively used in LinkHub, in particular named graphs [37] and Life Science IDentifiers or LSIDs [38]. Named graphs allow RDF graphs to be named by URI, allowing them to be described by RDF statements; named graphs could be used to provide additional information (metadata) about identifier mappings, such as source, version, and quality information. LSID is a standard object naming and distributed lookup mechanism being promoted for use on the Semantic Web, with emphasis on life sciences applications. An LSID names and refers to one unchanging data object, and allows versioning to handle updates. The LSID lookup system is in essence like what Domain Name Service (DNS) does for converting named internet locations to IP numbers. We could possibly use LSID for naming objects in LinkHub and incorporate LSID lookup functionality. Finally, like software such as Napster and Gnutella did for online file sharing, we plan to explore enhancing LinkHub to enable multiple distributed LinkHub instantiations to interact in peer-to-peer networks for dynamic biological data sharing, possibly using web services technologies such as Web Services Description Language (or WSDL) [39] and Universal Description, Discovery and Integration (or UDDI) [40] for dynamic service discovery, and available peer-to-peer toolkits.

## Conclusion

Our paper demonstrates the natural use of Semantic Web RDF to inter-connect identifiers of data entries residing in separate web-accessible biological databases. Based on such a semantic RDF graph of biological identifiers and their relationships, useful, non-trivial cross-database queries, inferences, and semantic data navigation can be performed through web interactive and query access. In addition, these semantic relationships enable flexible and novel information retrieval access based on queries of the LinkHub graph's relational structure to web documents attached to identifier nodes. LinkHub also can simplify and manage connections to major hubs such as UniProt for a lab or organization. LinkHub can be evaluated by considering its current active and practical use in a number of settings. We have already established the "hub of hubs" relationship between UniProt and LinkHub (i.e. UniProt cross-references to our LinkHub). In addition, LinkHub cross-references the targets of the structural genomics initiative to UniProt and serves as a "related links" and "family viewer" gateway for the Northeast Structural Genomics Consortium with which we are affiliated; LinkHub also serves as the "family viewer" for MolMovDB. LinkHub is a step towards answering the question "a life science Semantic Web: are we there yet?" [41].

## Methods

### Obtaining LinkHub data

A key problem in populating the LinkHub database (described below) is how to determine the relationships among biological identifiers, a specific case of the so-called ontology alignment problem [42,43]. Biology is blessed with a fundamental, commonly accepted principle around which data can be organized, namely biological sequences such as DNA, RNA, and protein, and various string matching techniques (such as dynamic programming [44] and BLAST [45]) for biological sequences can solve a large part of the ontology alignment problem in biology. LinkHub thus takes advantage of biological sequence matching, in particular conservative, exact sequence matching, to cross-reference or align biological identifiers. LinkHub also takes advantage of available sources of pre-computed identifier mappings, with the most important one being UniProt which is arguably the most important major proteomics resource and serves as LinkHub's backbone content (i.e. most relationships between identifiers in LinkHub are indirect through UniProt). The general strategy for mapping identifiers in LinkHub is to first take advantage of known and trusted pre-computed identifier mappings; if such pre-computed mappings are unavailable, an attempt is made to map identifiers based on exact sequence matches of their underlying sequences to UniProt and other sources of sequence data whose identifiers are stored in LinkHub.

Efficient, exact sequence matching programs were developed and used to do quick inter-database cross-referencing or alignment based on exact sequence matches (e.g. to cross-reference TargetDB to UniProt, see below). A custom Perl module was developed and used to index UniProt (and in general sequence databases in FASTA format [46]) to support this fast exact sequence matching. Specialized Perl web crawlers and other scripts were written to fetch and extract data from different sources in different formats; identifiers, identifier relationships, and other related information were extracted from the sources and inserted into the LinkHub MySQL database (which is also converted to RDF and inserted into the RDF version of LinkHub; see below). A running instantiation of the LinkHub system is at  and , and it is actively used and populated with data from the Gerstein Lab [47] and related to the lab's research interests. Thus while the ideas of LinkHub can be applicable more generally to biological data, the concrete instantiation of LinkHub focuses heavily on proteomics data, as that is a key research initiative of the Gerstein Lab. The "hub of hubs" relationship described above has already been established between UniProt and LinkHub (i.e. UniProt hyperlinks to the LinkHub instantiation and cross-references to it in its DR lines). In addition, LinkHub cross-references the proteins which are targets of the structural genomics initiative (obtained from the TargetDB resource [48]) to UniProt and the LinkHub instantiation serves as a "related links" and "family viewer" (more below) gateway for the Northeast Structural Genomics Consortium (NESG) [49] with which the Gerstein Lab is affiliated. Additional focuses of the LinkHub instantiation are yeast resources, macromolecular motions [13], and pseudogenes [50].

### LinkHub database models

LinkHub is conceptually based on the Semantic Web (graph) model and we thus represent it and store it in RDF. RDF is a popular data model (or ontological language) for the Semantic Web that represents data as a directed labelled graph. Essentially, in RDF URIs [51] are used for globally unique naming of the nodes (which represent objects) and the edges (which represent relationships between nodes) of the graph, and literal values may also be used in place of pointed to nodes. In addition, RDF comes with query languages (e.g., RDQL [52]) to allow the user to pose semantic queries against graph data. While there are more advanced ontological languages such as the Web Ontology Language or OWL [53] that support data reasoning based on Description Logics or DL [54], RDF is easy to learn and use and much can be effectively modelled with it. For example, the benefits of representing proteomics data in RDF were discussed [9] and UniProt data has also recently been made available in RDF format [55]. However, there could be a potential problem in performance and scalability when using the new RDF database technology, which can be an important impediment to more active and widespread use of the Semantic Web. In this regard, the creation of high-performance RDF databases should be a research priority of the Semantic Web community. Thus, while we would ideally use only RDF, to support LinkHub's practical daily use for its web interactive interfaces we also model and store its data using relational database technology (MySQL) for efficiency and robustness. A drawback is that relational databases do not naturally model graph structures or provide efficient graph operations for which special procedural codes are necessary (e.g. for the "path type" view described below). It is straightforward mapping between the relational and RDF versions of LinkHub and we have written Java code to do this.

**Figure 4:**
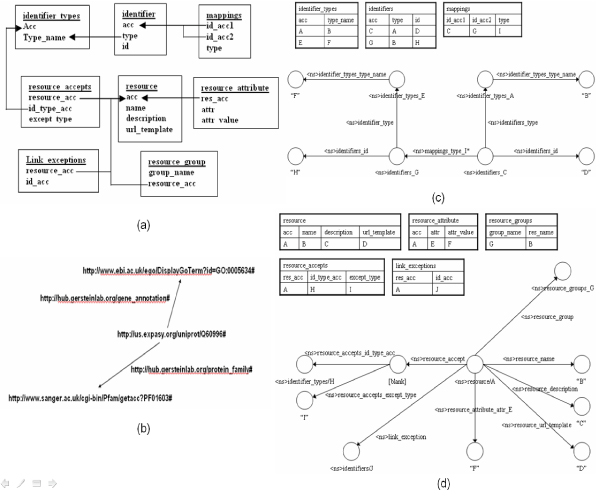
**LinkHub relational and RDF data models**. (a) LinkHub relational model (b) An example RDF graph of two statements or triplets: (Q60996, gene_annotation, GO:0005634) and (Q60996, protein family, PF01603), which describe that there is a protein (Q60996) whose gene annotation is identified by GO: 0005634 and protein_family by PF01603. This also exemplifies using an RDF graph to connect multiple resources. Here, it connects UniProt, Gene Ontology, and Pfam. The detailed description associated with each identifier can be provided by the corresponding resource (the URL or URI can provide access to such detailed descriptions). (c) Illustrates how the key LinkHub relational tables identifier_types, identifiers, and mappings (reproduced above the RDF structure) are mapped to the corresponding RDF structure. The resulting RDF graph captures different types of object identifiers stored in different databases and the relations (or mappings) between these object identifiers. The mapping types are explicitly represented as RDF properties. (d) Shows how the rest of the LinkHub relational tables (reproduced above the RDF structure) map to the RDF structure. The resulting RDF graph captures the different web resources (which can be grouped) accessible by LinkHub. In addition, the graph captures information about which web resources accept which types of object identifiers, as well as exceptions.

The relational structure of LinkHub, shown in figure 4a, reflects how the graph of biological identifier relationships and associated data, such as URLs of identifier-specific web pages, are managed and stored. Biological identifiers are stored in the identifier table and are typed, where the identifier_types table gives the type. Thus, for example, two different identifiers in separate databases which happen to have the same identifier text can nevertheless be distinguished by differing identifier types (based on the databases they come from). The mappings table is used to store the relationships between identifiers, with the "type" attribute giving the description or meaning of the relationship. The identifier table thus gives the nodes and the mappings table the edges of the graph of biological identifier relationships. The resource, resource_accepts, and link_exceptions tables together manage and store URLs for identifier-specific web pages (e.g. the web page at UniProt giving specific information particular to some UniProt identifier). The basic idea is that web resources such as UniProt have template URLs which can be interpolated with particular identifiers to generate identifier-specific URLs. The resource table contains a short name, longer description, and the template URL of web resources such as UniProt. The resource_accepts table lists the particular types of identifiers that can be interpolated into a resource's template URL, as well as an exception type except_type. The exception type is to handle cases where not all identifiers of an accepted type are legal, i.e. some of the identifiers cannot be interpolated into the template URL to generate a valid URL. If except_type is NONE then there are no exceptions and all identifiers of the type are accepted. Otherwise except_type has value NACC or ACC. If except_type is NACC, then the exceptions are explicitly given in the link_exceptions table (i.e. the identifiers in the link_exceptions table of the given type for the resource are the ones that cannot be interpolated into the template URL, and all other identifiers of the type CAN be interpolated). If except_type is ACC then the behaviour is the opposite: the identifiers NOT listed in the link_exceptions table are the exceptions and the ones explicitly listed are the only ones that can be interpolated into the resource's template URL. NACC and ACC exception types are both supported to allow the most efficient handling of exceptions, i.e. whichever is smaller between the set of accepted identifiers and the set of exception identifiers can be listed in link_exceptions thus minimizing the amount of space necessary for storing exceptions. The resource_group table supports grouping of web resources, e.g. all web resources maintained by the Gerstein Lab or relating to protein structure. Finally, the resource_attribute table allows free text attributes to be associated with web resource, however it is not currently used. Figure 4 also provides details of the LinkHub RDF model and how it is related to the relational model; a simple example RDF graph is also given.

### LinkHub web interfaces

The primary interactive interface to the LinkHub database is a web-based interface (implemented using the so-called AJAX technologies [56], i.e. DHTML, JavaScript, DOM, CSS, etc.) which presents subsets of the graph of relationships in a dynamic expandable/collapsible list view. This interface allows viewing and exploring of the transitive closure of the relationships stemming from a given identifier interactively one layer at a time: direct edges from the given identifier are initially shown and the user may then selectively expand fringe nodes an additional layer at a time to explore further relationships (computing the full transitive closure is prohibitive, and could also cause the user to "drown" in the data, and we thus limit it initially, and in each subsequent expansion, to anything one edge away, with the user then guiding further extensions based on which relationships he would like to explore). Figure 2 is a screenshot of the interface and gives more details of it. The second, "path type" interface presents results the same as the first interface (i.e. dynamic expandable/collapsible list view) but allows users to query and view particular identifier type paths in the LinkHub graph. For example, one might want to view all proteins in some database in the same Pfam family as a given protein; in LinkHub Pfam relationships are stored for UniProt proteins, so one could view the fellow family members of the given protein by specifying to view all relational paths in the LinkHub graph whose identifier types match:

Given protein in database → equivalent UniProt protein → Pfam family → UniProt proteins → other equivalent proteins in database.

## Competing interests

The authors declare that they have no competing interests.

## Authors' contributions

AKS is the primary (first) author and is responsible for the majority of the work, implementation, and writing. KYY did the conversion of the relational (MySQL) version of LinkHub to RDF, integrated it into YeastHub, and wrote and executed the demo RDF queries over the joined YeastHub/LinkHub. KHC, MS, and MBG are faculty advisors and provided high-level direction and guidance to the work.
